# Adjuvant nonavalent HPV vaccination in women treated for vulvar HSIL, a randomized placebo-controlled trial; VulVaccin study protocol

**DOI:** 10.1186/s12885-025-14275-w

**Published:** 2025-05-20

**Authors:** Vera J. G. M. Vaessen, Ralf L. O. van de Laar, Marta Piso-Jozwiak, Virgil A. S. H. Dalm, Elmar A. Joura, Matthias Jentschke, Heleen J. van Beekhuizen

**Affiliations:** 1 Department of Gynaecological Oncology, ErasmusMC Cancer Institute, University Medical Center Rotterdam, Rotterdam, 3015 GD The Netherlands; 2 Department of Internal Medicine, Division of Allergy & Clinical Immunology, Department of Immunology, Erasmus University Medical Center Rotterdam, Rotterdam, 3015 GD The Netherlands; 3 Department of Gynecology and Obstetrics, Comprehensive Cancer Center, the Medical University of Vienna, BT86/E 01, Spitalgasse 23, Vienna, 1090 Austria; 4 Department of Gynecology and Obstetrics, Klinikum Würzburg Mitte, Würzburg, 97074 Germany

**Keywords:** Vulvar neoplasms, Vulvar high-grade squamous intraepithelial lesion, Papillomavirus vaccines, Adjuvant HPV vaccination, Vulvar HSIL treatment, Vulvar cancer

## Abstract

**Background:**

Each year, 45,000 women worldwide develop vulvar cancer, often occurred from vulvar high-grade squamous intraepithelial lesions (vHSIL), a precancerous stage associated with high- risk human papillomavirus (HPV). Recurrent vHSIL, experienced by over 30% of patients despite treatment, conducting in significant physical and psychosocial challenges. With no established method to reduce recurrence, our study investigates the potential of adjuvant HPV vaccination during treatment. We aim to determine whether nonavalent HPV vaccination can effectively prevent vHSIL recurrence in women treated for vulvar HSIL.

**Methods:**

This is a randomized, double-blind, placebo-controlled study protocol involving 498 women diagnosed with vHSIL. Participants will be randomized to receive either the nonavalent HPV vaccine or a placebo in addition to standard treatment. The primary outcome is the recurrence of vHSIL within 24 months following treatment. Secondary endpoints are treatment effectiveness, immune response, cost-effectiveness, and quality of life. Long-term follow-up examines vaccine effect after 5 and 10 years, along with the occurrence of vulvar malignancies. Relative risk between vaccinated and placebo groups will be evaluated, employing intention-to-treat principles.

**Discussion:**

This study is designed to investigate the potential benefits of HPV vaccination in managing vHSIL. Results from the trial will provide evidence on the vaccine’s impact on recurrence rates, treatment outcomes, and long-term prevention of vulvar malignancies.

**Trial registration:**

ClinicalTrials.gov. Identifier: NCT06052696. Registered 12 January 2023, https://clinicaltrials.gov/study/NCT06052696.

## Background

Annually, approximately 45,240 women globally are affected by vulvar carcinoma, with a yearly mortality of 15.000 [1]. Around one-third of vulvar cancers arise from a precursor caused by high-risk Human Papilloma Virus (hrHPV), called vulvar High-Grade Squamous Intra-Epithelial Lesion (vHSIL). The standardized incidence rate of vHSIL in Europe is estimated to be approximately 3.3 per 100,000 women years. In the Netherlands, an average of 300 women are newly diagnosed with vHSIL annually. Across Europe, there has been a consistent upward trend in vHSIL incidence over the past two decades, mirroring patterns observed in HPV-related malignancies [1]. The peak incidence of these precancerous vulvar lesions typically occurs between the ages of 35 and 40 [2]. Approximately 10% of women diagnosed with vHSIL, despite undergoing treatment, will progress to develop vulvar cancer within ten years after initial diagnosis. Recurrent vHSIL substantially increases the risk of malignancy [2].

Despite available treatments such as surgery, laser vaporization, and topical imiquimod, vHSIL recurrence rates remain high, affecting at least 30% of treated women within two years, regardless of treatment modality [2, 3]. Unfortunately, effective preventive strategies to address vHSIL recurrence are currently absent.

Promising empirical evidence suggests that HPV vaccination could reduce the risk of vHSIL recurrence. A retrospective secondary analysis of pooled data of two RCTs showed a significant reduction of 35% (irrespective of causal HPV type) to 64% (related to vaccine type) for recurrences of vHSIL and low-grade squamous intraepithelial lesion (LSIL) after adjuvant quadrivalent HPV vaccination [4]. Recently, a case-control study was published showing a reduction of 40% in recurrences after quadrivalent HPV vaccination; due to small groups, this reduction was not significant [5]. There is a notable absence of high-quality studies investigating adjuvant nonavalent vaccination, specifically in women treated for vHSIL.

We propose a prospective, double-blind, randomized controlled trial to evaluate the efficacy of adjuvant nonavalent HPV vaccination compared to placebo in reducing recurrence rates among women currently treated for vHSIL. Given that almost all HPV variants linked to vHSIL are covered by the nonavalent vaccine, this trial seeks to provide a novel preventive strategy for vHSIL recurrence [6–9].

This study aims to investigate the efficacy of nonavalent HPV vaccination versus placebo in preventing recurrence in women treated for vHSIL.

## Methods

### Study design

**Fig. 1:**
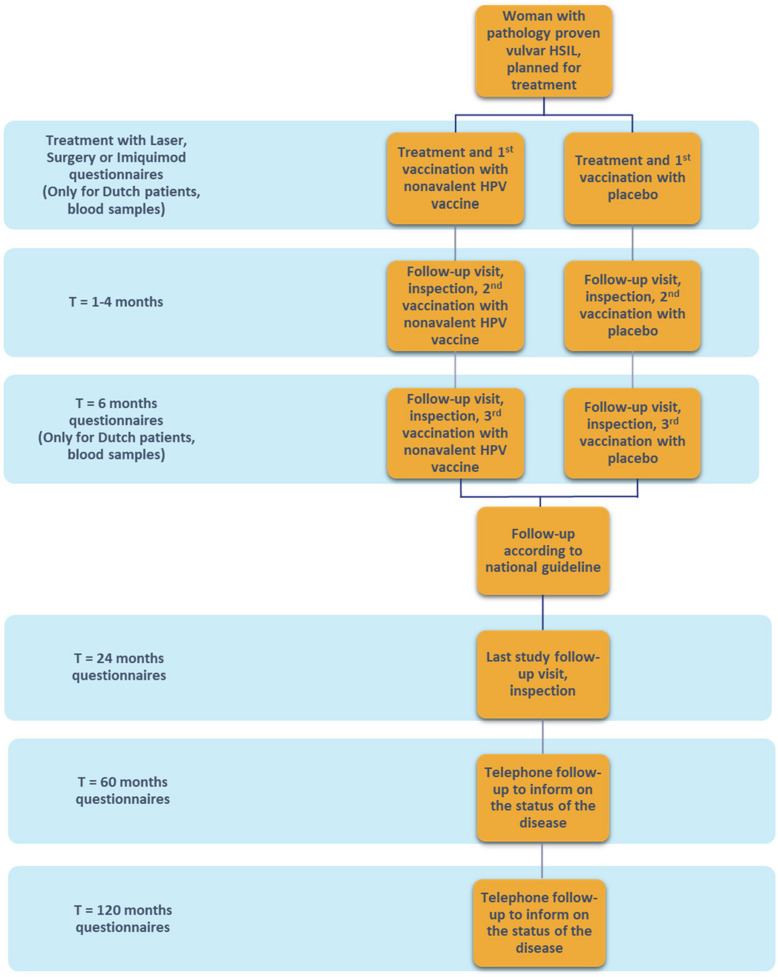
Study design

This is a randomized, multicenter, double-blinded, placebo-controlled trial involving 498 women diagnosed with vHSIL and scheduled for receiving treatment. Eligible participants will include women with histologically diagnosed vHSIL, both primary lesions and those with recurrence. Treatment follows current guidelines. Participants will be randomized to either receive adjuvant HPV vaccination or a placebo during treatment initiation, approximately two and six months into treatment (three dose regimen). All participants will undergo a two-year clinical follow-up as part of the trial. Throughout the study, participants will complete questionnaires, with Dutch participants optionally undergoing blood sampling to assess immune response three times. Participants may opt in to the blood sampling procedure, which is not obligatory for trial participation. Follow-up assessments adhere to current guidelines, and patients will receive standard treatment per the latest vHSIL guidelines. Long-term follow-up will examine vaccine efficacy after 5 and 10 years, along with the occurrence of vulvar malignancies (Figure 1 in Appendix).

### In- and exclusion criteria

Women aged eighteen or above with histologically proven vHSIL and planned for treatment are eligible for participation. Exclusion criteria include prior HPV vaccination, current invasive carcinoma or history of HPV-related genital carcinoma (cervix, anal, vulva), current pregnancy, allergy to vaccine components, patients with primary or secondary immunodeficiencies, including patients with HIV or on immunosuppressive medication, (see Tables 1 and 2 in the Appendix) and Human Immunodeficiency Virus (HIV) infection.

### Recruitment

Patients will be counseled about the study during consultations at the participating clinics. They will receive patient information along with an informed consent form, either on paper or digitally. If the histologic diagnosis is vHSIL and treatment is scheduled, the patient is eligible to enroll in the study.

### Study objectives and endpoints

The primary objective is to evaluate the efficacy of adjuvant nonavalent HPV vaccination in women who will undergo treatment for (histologically proven) vHSIL in preventing clinical recurrence of vHSIL up to 24 months.

The second objectives are:

1. The effectiveness (complete remission) after treatment in patients receiving vaccination versus placebo at 6 and 12 months.
2. Complete remission after treatment in patients receiving vaccination versus placebo at 6 and 12 months in primary episode (newly diagnosed) vHSIL versus recurrence.
3. The effectiveness of adjuvant vaccination in different treatments of vHSIL (laser, imiquimod, excision) after 24 months.
4. Additional treatment for vHSIL needed in the study period, and differences between the study groups.
5. The effect of vaccination versus placebo on different hrHPV types (HPV type of primary and recurrence).
6. The difference in immune response to HPV vaccination versus placebo vaccination. (We use HPV antibodies as a surrogate marker for immune response. Peripheral blood mononuclear cells (PBMCs) are bio banked for the possibility to measure in-depth cellular immune response at the molecular level at the end of the trial).
7. Cost-effectiveness (IMTA Medical Consumption Questionnaire (iMCQ) and IMTA Productivity Cost Questionnaire (iPCQ) will be used).
8. Quality of life (measured by Euroqol 5D-5L questionnaire) and sexual health (measured by Female Sexual Function Index, FSFI questionnaire) improved after vaccination compared to placebo.
9. The effect of vaccination versus placebo on CIN/cervical cytology of the uterine Cervix.
10. Long-term follow-up: The difference between HPV vaccination and placebo group in number of recurrences of vHSIL (5 and 10 years) and vulvar malignancies (2,5 and 10 years).

The primary endpoint is a difference in the number and percentage of patients with a clinical recurrence rate of vHSIL between HPV vaccination and placebo up to 24 months post-treatment.

The secondary endpoints are:

1. Difference in the number and percentage of patients with a clinical recurrence rate of vHSIL between HPV vaccination and placebo at 6 and 12 months.
2. Difference in the number and percentage of patients with a clinical recurrence rate of vHSIL between HPV vaccination and placebo at 6 and 12 months for primary vHSIL versus recurrent vHSIL.
3. Difference in the number and percentage of patients with a clinical recurrence rate of vHSIL between the different treatment modalities.
4. Difference in number and percentage for additional treatment necessary after primary treatment.
5. Description and percentage of different hrHPV types. Percentage clearance of primary HPV type for vaccination versus placebo.
6. Concentration of HPV antibodies and percentage of increase or decrease after vaccination.
7. Is there a correlation between the number or percentage of recurrence? Is that different in primary versus recurrence episodes of vHSIL.
8. Incremental cost-effectiveness ratio (ICER) describes the difference in costs and budget impact analysis.
9. Description and change in numeric value and percentage in different score forms questionnaires. Percentage increase of decreased quality of life, sexual impact, and productivity impact.
10. Difference in number and percentage of HPV types in cervical cytology and vHSIL before and after vaccination.
11. Difference in number and percentage in the recurrence rate of vHSIL and vulvar cancer between HPV vaccination and placebo. Other HPV-related diseases known?

### Data collection

All data will be collected using an electronic Case Report Form (eCRF) and electronic questionnaires. Data from the eCRF and questionnaires will be collected and stored using Castor EDC. Sequential subject numbers will be assigned to enrolled participants in the study. Access to stored data will be restricted solely to the principal and coordinating investigators of each participating center.

Demographic and baseline disease characteristic data will be summarized for each treatment group and consist of age, parity, cervical cytology and HPV status, prior treatment for vHSIL, prior treatment for cervical HSIL, smoking, and co-morbidities in which we focus on auto-immune diseases and history of cancer.

At each follow-up visit, the following data will be collected: visual/physical examination and preferably photo documentation of the vulva. If there is any suspicion of recurrence of vHSIL, a biopsy will be taken for histology and HPV typing endpoint, cervical cytology, and if additional treatment will be started.

At the start of the study, after 6, 24, and 60 months, patients will be asked to fill out questionnaires.

### Materials and equipment

The adjuvant HPV vaccine to be administered is the nonavalent HPV vaccine, which is globally approved and utilized in national immunization programs for children. The nonavalent HPV vaccination is an adjuvant non-infectious recombinant 9-valent vaccine. It is prepared from the highly purified virus-like particles (VLPs) of the major capsid L1 protein from the same four HPV types (6, 11, 16, 18) in the qHPV vaccine Gardasil or Silgard and five additional HPV types (31, 33, 45, 52, 58). It uses the same amorphous aluminum hydroxyphosphate sulfate adjuvant as the qHPV vaccine. The VLPs cannot infect cells, reproduce, or cause disease. The efficacy of L1 VLP vaccines is thought to be mediated by the development of a humoral immune response.

The placebo consists of physiological saline (NaCl) with no active ingredients. Preparation and labeling of the investigational medicinal products will be done according to the relevant GMP guidelines. The labels will be in Dutch for the Dutch centers (according to the CCMO rules) and will be translated into the language of each participating country. Distribution will be organized by the Erasmus MC Pharmacy.

### Detailed procedure

Patients with vulvar lesions are referred to the gynecologist according to their regular care. In the case of vHSIL, a biopsy will be taken for histological examination by a pathologist as part of regular care. At that time, potential participants will receive oral and written study information. In the case of histology-proven vHSIL participants will be invited for the screening visit.

In cases of uncertainty about whether a lesion is primary, residual, or recurrent, we have established an independent board of three experts on vulvar diseases to provide a definitive decision by consensus.

During the screening visit, routine cervical cytology (if not previously taken) and HPV status are obtained, along with visual examinations, including photo documentation to monitor lesion progression or regression.

At the follow-up visits, patients will undergo visual and physical examinations, with a preference for photo documentation of the vulva, to monitor any changes or recurrence of lesions or the development of new lesions. In case of suspicion of recurrence or a new lesion, a biopsy is taken. When it is not feasible to take a new biopsy, but new treatment is initiated, this is also considered a clinical recurrence. Quality-of-life questionnaires and immune response analysis will be performed, adhering to guideline-based care.

Recurrence is defined as any reappearance of vHSIL after six months from the start of treatment, while residual disease is defined as the presence of clinical suspicion and preferable histological conformation of vHSIL within six months from the start of the treatment.

In case of uncertainty regarding the histological diagnosis, an expert gynecological pathologist will review the slides digitally.

### Randomization, stratification, blinding and treatment allocation

Randomization will be performed through the secure web-based interface Castor EDC (Castor Electronic Data Capture available at: https://castoredc.com). Patients will be randomly assigned to either adjuvant HPV vaccination or placebo in a 1:1 ratio. Randomization will be stratified by country of inclusion, treatment group (surgery, laser vaporization, and topical imiquimod), and the first episode of vHSIL versus recurrence. The study team, the local investigator, and the patient are blinded for treatment allocation of the study medication. The allocated medication in the Netherlands will be prepared by the pharmacy at the Erasmus MC and administered to the patient by a designated study team member from Erasmus MC. Study medication will be masked for the administrator of the vaccination and the patient.

### Interventions

Patients eligible for participation will be randomized to either a nonavalent HPV vaccine or a placebo vaccine (physiological salt solution for injections). The follow-up will follow the European College for the Study of Vulval Disease (ECSVD) guideline for vHSIL.

The first vaccination is preferably administered concurrently with treatment initiation (utilizing either surgery, laser vaporization, or topical imiquimod). This window spans four weeks prior to treatment commencement (due to potential logistical considerations regarding surgical scheduling) and four weeks post-treatment initiation. The second vaccination should be administered at least one month after the first vaccination and three months preceding the third vaccination. The third vaccination should be administered at least three months after the second vaccination. All vaccinations must be administered within six months.

### Sample size calculations

The sample size calculations are based on the available evidence, which describes a recurrence rate of 19% versus 32% in favor of HPV vaccination [5]. With a target power of 0.8 and a two-sided alpha of 0.05, we need a total of 498 patients (249 in each arm), including a loss of follow-up and a 20% correction of the supposed different effect in treating the first episode of vHSIL versus the recurrence of vHSIL.

### Data analysis

The primary outcome will assess the relative risk of vHSIL recurrence within 24 months, analyzing the efficacy of adjuvant nonavalent HPV vaccination using logistic regression methods adjusted for country of inclusion, treatment types, and primary versus recurrence vHSIL. A 95% confidence interval will be estimated for the odds ratio, with significance determined by a likelihood ratio test. *P*-values below 0.05 will be considered statistically significant.

Analysis of secondary endpoint(s) Secondary objectives 1, 2, 4, 9, and 10 will be analyzed similarly to the primary endpoint, using logistic regression methods adjusted for the stratification variables: country of inclusion, treatment types, and primary versus recurrence vHSIL. 95% Confidence intervals will be estimated for the odds ratios and significance will be determined using likelihood ratio tests. *P*-values below 0.05 will be considered statistically significant.

Secondary objectives where the effectiveness of vaccination is compared between different patient groups (secondary objectives 3 and 5) will be analyzed using logistic regression methods adjusted for stratification variables. 95% Confidence intervals will be estimated for the odds ratio, and significance will be determined using a likelihood ratio test. *P*-values below 0.05 will be considered statistically significant.

Secondary objective 6 will be analyzed using a linear regression model to determine the difference in log-transformed antibody levels at baseline and after placebo or vaccination and adjusted for stratification variables. The impact of change in antibody levels on the primary endpoint will be analyzed using a logistic regression model adjusted for stratification variables. 95% Confidence interval will be estimated for the coefficients, and significance will be determined using a likelihood ratio test. *P*-values below 0.05 will be considered statistically significant.

Secondary objective 8: Quality of Life will be measured at several time points. They will be assessed with a Euroqol 5D-5L questionnaire. Sexual health will be evaluated using FSFI. The impact of vaccination on these measures over time will be analyzed using linear mixed-effect models, with vaccination status and stratification variables as fixed effects and random intercepts as random effects. Additional random effects will be selected based on likelihood ratio tests. A 95% Confidence interval will be estimated for the coefficients, and significance will be determined using a likelihood ratio test. *P*-values below 0.05 will be considered statistically significant.

Secondary objective 7: Cost-effectiveness will be assessed by calculating the incremental cost-effectiveness ratio (ICER), which is defined as the difference in costs divided by the average change in the effectiveness of the vaccine versus placebo following vHSIL treatment. A Budget impact analysis (BIA) will be performed, in accordance with the principles for good practice for BIA, to address the financial stream of consequences to assess the affordability of offering the vaccine following vHSIL treatment.

#### Expected results

Given the available evidence, it is anticipated that adjuvant HPV vaccination in patients treated for vHSIL may have a beneficial impact. We hypothesize that adjuvant HPV vaccination will show effectiveness in reducing the recurrence of vHSIL and could potentially decrease the incidence of vulvar cancer. If the vaccine is proven to be successful in reducing recurrent vHSIL, we will implement adjuvant vaccination in the guidelines for insurance reimbursement.

## Data Availability

No datasets were generated or analysed during the current study.

## Appendix

Consists of the following tables and figures; Figure 1, Table 1, and Table 2

**Table 1:** Allowed medication(s)

**Generic Name**	**Brand Name**	**Class of Drug**
Adalimumab	Humira	TNF-blocker
Budesonide (≤5 mg)	Entocort EC	Corticosteroid
Cetroluzimab	Cimzia	TNF-blocker
Dexamethasone (≤2.5 mg/day)	Dexamethason	Corticosteroid
Etanercept	Enbrel	TNF-blocker
Golimumab	Simponi	TNF-blocker
Hydrocortisone (≤60 mg/day)	Hydrocort	Corticosteroid
Hydroxychloroquine	Plaquenil	DMARD
Infliximab	Remicade	TNF-blocker
Prednisolone (≤15 mg/day)	Pediapred	Corticosteroid
Prednisone (≤15 mg/day)	Deltasone	Corticosteroid

**Table 2:** Not-allowed medication(s)

**Generic Name**	**Brand Name**	**Class of Drug**
5-fluorouracil	Carac	Antimetabolite
Abatacept	Orencia	Immunomodulator
Altretamine	Hexalen	Alkylating Agent: Aziridines and Epoxides
Anakinra	Kineret	Biological immunosuppressive agent
Azathioprine	Imuran	Transplant immunosuppressive agent
Basiliximab	Simulect	Transplant immunosuppressive agent
Belatacept	Nulojix	Transplant immunosuppressive agent
Bendamustine	Bendeka	Alkylating agent: Nitrogen mustard
Busulfan	Myleran	Alkylating agent: Alkyl sulfonate
Carmustine	Gliadel Wafer	Alkylating agent: Nitrosourea
Chlorambucil	Leukeran	Alkylating agent: Nitrogen mustard
Cisplatin	Platinol	Alkylating agent
Cyclophosphamide	Nerosar	Alkylating agent: Nitrogen mustard
Cyclosporine	Gengraf, Neoral	Calcineurin inhibitor
Cytarabine	Cytosar-U	Antimetabolite
Diaziquone	Proglycem	Alkylating agent: Aziridine and Epoxide
Everolimus	Zortress	mTOR inhibitor
Ixekizumab	Taltz	Other biological agent
Ifosfamide	Ifex	Alkylating agent: Nitrogen mustard
Lomustine	Gleostine	Alkylating agent: Nitrosourea
Mechlorethamine	Valchlor	Alkylating agent: Nitrogen mustard
Melphalan	Evomela	Alkylating agent: Nitrogen mustard
Mercaptopurine	Purinethol	Antimetabolite
Methotrexate	Otrexup, Trexall,	Antimetabolite
Mycophenolate mofetil	CellCept	Transplant immunosuppressive agent
Natalizumab	Tysabri	Other biological agent
Oxaliplatin	Eloxatin	Alkylating agent
Procarbazine	Matulane	Alkylating agent
Rituximab	Rituxan	Monoclonal antibody
Secukinumab	Cosentyx	Other biological agent
Sirolimus	Rapamune	mTOR inhibitor
streptozocin	Zanosar	Nitrosourea
Tacrolimus	Protopic,	Calcineurin Inhibitor
temozolomide	Temodar	Alkylating agent
Thioguanine	Tabloid	Antimetabolite
thiotepa	Tepadina	Alkylating agent
Tocilizumab	Actemra	Immunosuppressive agent
Tofacitinib	Xeljanz	Janus kinase inhibitor
Ustekinumab	Stelara	Monoclonal antibody
Vedolizumab	Entyvio	Monoclonal antibody

The prohibited medication only applies at the time of inclusion. When a patient receives one of these medications during the study, the subject will remain in the trial

## Abbreviations

HPV: Human papillomavirus
vHSIL: Vulvar High-grade squamous intraepithelial lesions
hrHPV: High-risk Human Papilloma Virus
LSIL: Low-grade squamous intraepithelial lesion
VulVaccin: Acronym for Adjuvant nonavalent HPV vaccination in women treated for vulvar HSIL
HIV: Human Immunodeficiency Virus
PBMCs: Peripheral blood mononuclear cells
IMTA: Institute for Medical Technology Assessment
iPCQ: IMTA Productivity Cost Questionnaire
iMCQ: IMTA Medical Consumption Questionnaire
Euroqol 5D-5L: European Quality of Life 5 Dimensions 5 Level Version
FSFI: Female Sexual Function Index
CIN: Cervical Intra-epithelial Neoplasia
eCRF: Electronic Case Report Form
Castor EDC: Castor Electronic Data Capture
VLPs: Purified virus-like particles
qHPV: Quadrivalent
NaCl: Physiological saline
GMP: Good Manufacturing Practice
CCMO: Central Committee on Research Involving Human Subjects
ErasmusMC: Erasmus Medical Centre
ECSVD: European College for the Study of Vulval Disease
ICER: The Incremental cost-effectiveness ratio
BIA: Budget impact analysis

## References

[1] Sung H, Ferlay J, Siegel RL, Laversanne M, Soerjomataram I, Jemal A, Bray F. Global cancer statistics 2020: GLOBOCAN estimates of incidence and mortality worldwide for 36 cancers in 185 countries. CA Cancer J Clin. 2021;71:209–49. 33538338 10.3322/caac.21660

[2] Thuijs NB, Beurden MV, Bruggink AH. Vulvar intraepithelial neoplasia: incidence and long-term risk of vulvar squamous cell carcinoma. J Cancer. 2021;148:90–8. 10.1002/ijc.33198 PMC7689827 32638382

[3] Bryan S, Barbara C, Thomas J, Olaitan A. HPV vaccine in the treatment of usual type vulval and vaginal intraepithelial neoplasia: a systematic review. BMC Womens Health. 2019;19:3. 30616555 10.1186/s12905-018-0707-9 PMC6323700

[4] Joura EA, Garland SM, Paavonen J, Ferris DG, Perez G, Ault KA, et al. Effect of the human papillomavirus (HPV) quadrivalent vaccine in a subgroup of women with cervical and vulvar disease: retrospective pooled analysis of trial data. BMJ. 2012;344:e1401. 22454089 10.1136/bmj.e1401 PMC3314184

[5] Ghelardi A, Marrai R, Bogani G, Sopracordevole F, Bay P, Tonetti A, et al. Surgical treatment of vulvar HSIL: adjuvant HPV vaccine reduces recurrent disease. Vaccines (Basel). 2021;9(2):83. 10.3390/vaccines9020083 PMC7911252 33503866

[6] van der Avoort IA, Shirango H, Hoevenaars BM, Grefte JM, de Hullu JA, de Wilde PC, et al. Vulvar squamous cell carcinoma is a multifactorial disease following two separate and independent pathways. Int J Gynecol Pathol. 2006;25:22–9. 16306780 10.1097/01.pgp.0000177646.38266.6a

[7] Garland SM, Joura EA, Ault KA, Bosch FX, Brown DR, Castellsague X, et al. Human papillomavirus genotypes from vaginal and vulvar intraepithelial neoplasia in females 15–26 years of Age. Obstet Gynecol. 2018;132:261–70. 29995724 10.1097/AOG.0000000000002736

[8] Skapa P, Zamecnik J, Hamsikova E, Salakova M, Smahelova J, Jandova K, et al. Human papillomavirus (HPV) profiles of vulvar lesions: possible implications for the classification of vulvar squamous cell carcinoma precursors and for the efficacy of prophylactic HPV vaccination. Am J Surg Pathol. 2007;31:1834–43. 18043037 10.1097/PAS.0b013e3180686d10

[9] BrusenVilladsen A, Bundgaard-Nielsen C, Ambuhl L, Tang Svendsen M, Sokilde Pedersen I, Staehr Hansen E, et al. Prevalence and type distribution of human papillomavirus infections in Danish patients diagnosed with vulvar squamous cell tumors and precursors. Gynecol Oncol Rep. 2021;37:100828. 34621943 10.1016/j.gore.2021.100828 PMC8484492

